# Advanced Mobile Communication Techniques in the Fight against the COVID-19 Pandemic Era and Beyond: An Overview of 5G/B5G/6G

**DOI:** 10.3390/s23187817

**Published:** 2023-09-12

**Authors:** Chin-Feng Lin, Shun-Hsyung Chang

**Affiliations:** 1 Department of Electrical Engineering, National Taiwan Ocean University, Keelung 20224, Taiwan; 2 Department of Microelectronics Engineering, National Kaohsiung University of Science and Technology, Kaohsiung 81157, Taiwan

**Keywords:** 5G, B5G, 6G, mobile applications, COVID-19, beyond

## Abstract

The coronavirus disease 2019 (COVID-19) pandemic has severely affected people’s lives worldwide in an unexpected manner. According to the World Health Organization (WHO), several viral epidemics continue to occur and pose a significant public health problem. Until May 2023, there have been 676 million cases of COVID-19 infections and over 6.8 million deaths, globally. This paper surveys the role and effectiveness of advanced fifth-generation (5G)/beyond 5G (B5G)/sixth-generation (6G) technologies, combined with mobile applications (apps) and the Internet of Medical Things (IoMT), in detecting, managing, and mitigating the spread of COVID-19 and designing smart healthcare infrastructures for future pandemics. Analyzing and summarizing the research of relevant scholars based on the impact of 5G/B5G/6G and other technologies on COVID-19. The study tabulates the technical characteristics and effectiveness of different technologies in the context of COVID-19, summarizing the research of previous scholars. Challenges and design issues in the implementation of advanced information and telecommunication systems were demonstrated. These technologies can inspire the design of smart healthcare infrastructures to combat future virus pandemics.

## 1. Introduction

The coronavirus disease 2019 (COVID-19) pandemic has been recorded as a novel coronavirus of typical pneumonia since 31 December 2019 by the World Health Organization (WHO). Until May 2023, there have been 676 million cases of COVID-19 infections and over 6.8 million deaths, worldwide. Major sectors including industry, economics, education, and medicine have been affected. Viral infections are a major public health concern [1]. The nature of rapid, widespread, and frequent variations increases the difficulty of precise COVID-19 prevention, detection, control, and treatment. In the era of the fifth generation (5G), beyond 5G (B5G), sixth generation (6G), medical cloud, mobile applications (apps), Internet of Medical Things (IoMT), and artificial intelligence (AI), advances in bioinformatics techniques have introduced unprecedented opportunities for virus informatics studies, which contribute to the systems-level modeling of virus biology. Translational applications of recently developed data-driven and AI-assisted methods to viral cases, such as those in the COVID-19 pandemic era and beyond, have been emphasized. 

5G is the next generation of mobile communications technology beyond the fourth-generation (4G) long-term evolution (LTE) [2]. While wireless voice telephony and wireless broadband data transmission remain the primary applications of mobile communications systems, new applications for the Internet of Things (IoT) and the fourth industrial revolution have begun to drive the future growth of mobile communications systems. 5G mobile communication systems incorporate advanced technological solutions to achieve higher data rates, lower latency, greater capacity, and more efficient spectrum utilization. The next-generation wireless access technology, New Radio (NR), can provide diverse usage scenarios and applications envisioned for the 5G era. In addition, 5G provides more efficient networks and enables new services, ecosystems, and revenues. As a new generation of cellular technology typically emerges every 8–10 years, 6G is expected to be developed by 2030 [3]. Research materials for the 6G advanced mobile communication program include antennas, software, and advanced multiple access schemes. 6G could offer high-fidelity holograms, multisensory communications, terahertz (THz) communications, and pervasive AI. The evolution from 5G to 6G was explored from service, air interface, and network perspectives.

Emerging technologies including advanced mobile communication and networks, IoMT, machine learning, and AI play an important role in various fields such as healthcare, economics, education, and medical systems to monitor or tackle the impact of the COVID-19 pandemic [4]. Emerging technologies and other associated technologies have a significant impact on virus detection, tracking, and the mitigation of the risk of community transmission. The constant monitoring of viral infection, quick diagnosis, treatment, observation of the mass gathering and containment zone, contact tracing, helping medical doctors and nurses, enabling advanced information and telecommunication, and providing continuous virtual e-learning, all of these requirements strongly rely on the availability of robust mobile communications. IoMT is one such technology wherein physical objects are embedded with sensors, software, smartphones, mobile apps, and advanced mobile and network connectivity; these objects can sense the outside world, process, interpret, and forecast real-time data, communicate, and exchange medical information through a mobile cloud network. The IoMT system should include data collection and transfer from infected patients, data analytics with AI, hospitals and quarantine, and healthcare functions in the medical domain. 

This has driven rapid changes in many healthcare fields during the COVID-19 pandemic. Telehealth refers to the use of information and telecommunication technologies to support remote health care across multiple disciplines. Telehealth was used to mitigate the risks and consequences of the disease during the COVID-19 pandemic [5]. This study aimed to map the research landscape into a coherent taxonomy and characterize this emerging field in terms of motivation, open challenges, and solutions. Telehealth applications with respect to control, technology, and medical procedures were demonstrated, and the full potential of telehealth schemes during the COVID-19 pandemic and beyond was revealed. The telehealth system provides a platform on which physicians and patients can interact, regardless of the time or day, using smartphones or webcam-enabled computers. These innovations have provided instructions on how to overcome COVID-19. Clear insights into the impact of telehealth on the COVID-19 pandemic and beyond were explored. Center-based cardiac rehabilitation (CR) programs were integrated into telehealth modes (smartphone, telephone, web-based, or online) of delivery in regional and rural Australia during the COVID-19 public health emergency. Clinical guidelines recommend that all patients with acute coronary syndrome are important, with their self-care and self-prevention views increasing, and telehealth services facing an increased demand [6]. Green et al. [7] described the rapid deployment of a telehealth system with real-time (RT) video conference on chiropractic services in response to COVID-19. Musculoskeletal telehealth services include examinations, risk assessments, advice, and rehabilitative exercises, which were quickly developed to continue chiropractic care for patients. The patients reported that the appointments were helpful, addressed their concerns, and provided a safe method of seeing their doctors during the COVID-19 pandemic.

5G telecommunication networks, IoMT, and data analysis methods with machine-learning algorithms are currently used in different areas of health science, epidemiology, pharmacy, and virology to overcome the damage caused by pathogens [8]. Coronavirus is a respiratory illness that affects breathing patterns and other vital parameters. Some of the most distinguishing characteristics of COVID-19-positive patients are their breathing when they speak, a dry cough, and their breathing patterns. The AI algorithm identifies coughs and human respiratory sound recognition systems can analyze a person’s voice and provide a score regarding the likelihood of an individual having coronavirus. There is a growing need for more efficient and innovative methods to collect, process, analyze, and interpret massive and complex data [9]. An overview of challenges in big data problems and how innovative analytical methods, AI tools, and metaheuristics can tackle general healthcare problems with a focus on the current COVID-19 outbreak is provided. Modern digital technology, statistical methods, data platforms, and data integration systems to improve the diagnosis and treatment of diseases in clinical research and novel epidemiologic tools to tackle infection source problems are presented. Analyzing and interpreting medical data is a highly challenging task that requires multi-disciplinary efforts to continuously create more effective methodologies and tools to transfer clinical data information into knowledge that enables informed decision-making. Mobile telemedicine involves the use of advanced, ultra-low-latency, and reliable communication techniques to deliver real-time biomedical signals to patients at any place and time [10]. Mobile telemedicine adopts advanced concepts and techniques from the fields of electrical engineering, computer science, biomedical engineering, and medicine to overcome the restrictions of conventional medicine and improve its quality of service. Several mobile telemedicine systems have been illustrated, and it is important to gain a good understanding of mobile telemedicine systems because such systems are expected to become ubiquitous for the delivery of biomedical signals to patients and medical personnel for medicine. Hilbert–Huang transformation (HHT) is one of the principal time-frequency feature extraction methods for biomedical signals [11]. HHT-based time-frequency feature extraction schemes for biomedical signals, such as electroencephalograms, electrocardiogram signals, electrogastrogram recordings, and speech signals are mentioned. The HHT-based analysis methods and system features of medical signal applications are discussed in detail. In our previous research works [12,13,14], feature analysis of spike waves in epilepsy, feature analyses of FP1, FP2, and Fz electroencephalogram (EEG) signals in alcoholism, and energy feature information of F5 and F6 movements and motor imagery EEG signals in delta rhythms were illustrated.

This paper discusses innovative communication approaches for tackling COVID-19-related problems using modern mobile communication technologies and mobile apps to transmit medical data and vital signals. An overview of advanced 5G/B5G/6G mobile communication techniques, medicine technology in the fight against the COVID-19 pandemic, and mobile telemedicine is presented in the introduction section. Several studies have summarized the impact of 5G/B5G/6G and mobile apps on the COVID-19 pandemic from various perspectives. The significant role of IoMT technologies in the COVID-19 pandemic consists of the following seven components: detection and diagnosis, controlling the spread of the virus, quarantine mobile tracking, contact tracking, automated industry, assisting health, m-commerce, and mobile learning education systems. An advanced 5G/B5G/6G mobile internet offers high-speed transmission, high coverage, low latency, and reliable effective connection characteristics. The question of how 5G/B5G/6G, mobile applications, and associated emerging technologies can be useful in dealing with the post-COVID-19 situation from different aspects is explored. This paper provides an in-depth overview of the role of 5G/B5G/6G, mobile applications, and other emerging technologies in the detection, identification, and reduction of the spread of COVID-19. Section 2 discusses 5G, B5G, and 6G communication approaches to address important COVID-19-related clinical research questions. Section 3 describes state-of-the-art mobile applications that can provide new insights into mobile platform designs for COVID-19 and beyond. Section 4 presents the discussions. Section 5 concludes the paper by emphasizing the importance of multidisciplinary research and the continuing central role of medical data transmission in the era of advanced mobile communications.

## 2. Advance 5G, B5G, and 6G Mobile Communication Technologies Related to COVID-19

5G and IoMT-related technologies can be efficiently utilized and developed to fight the COVID-19 pandemic [15]. These technologies can enable innovative solutions in the areas of real-time (RT) telehealth services (TS), contact tracing (CT), education, retail and supply chains, urging governments to implement immediate control policies (ICPs), remote offices, information sharing, smart manufacturing, factory automation, m-tourism, and entertainment along with their technical requirements and challenges. The 5G and IoMT-related technical requirements of use-cases for application, expected capacity, expected latency, number of devices, and other requirements are illustrated. The expected capacity is 30 Mbps to 100 Gbps; the number of devices is 1000 to 1 million per city; and the expected latency of end to end is less than 1 ms. The use of advanced technologies such as IoMT, unmanned aerial vehicles (UAVs), robots, smart wearable medical devices (SWMDs), blockchain (BC), AI, and 5G has been evaluated to combat the COVID-19 pandemic [16]. During the early stages of COVID-19, we found that numerous false reports and misinformation caused unnecessary panic. COVID-19 causes ailments, such as cough, fever, fatigue, and breathlessness. Most patients exhibit clinical characteristics, such as fever, dry cough, fatigue, sore throat, headache, myalgia, sputum production, and breathlessness. COVID-19 presents opportunities and challenges for the automotive, aviation, tourism, oil, construction, food, healthcare, medical, and telecommunication industries. The technical effectiveness of the 5G-based IoMT system includes combat and prevention COVID-19 strategies (CPCS), diagnosis, and treatment COVID-19 strategies (DTCS). The technology is a core fundamental infrastructure (CFI), and effectively reducing COVID-19 mortality rates (ERCMR) can be achieved. He et al. [17] demonstrated that a 5G-based mobile video visitation system with virtual reality (VR) can decrease the psychological problem of COVID-19 patients in the intensive care unit (ICU). A 5G-based mobile video visitation system with VR can help family members, who are in a state of confusion, better understand what is happening to the patient, while informing them of the many efforts made by the caregivers to help the patient recover from the disease. 5G healthcare systems include SWMDs, VR functions, smart hospital care, and remote diagnosis. Decreasing the psychological problems of ICU COVID-19 patients (DPPICUDP), CPCS, DTCS, CFI, and ERCMR can be achieved.

Moglia et al. [18] examined B5G-based cloud communication technologies primarily on the major components of healthcare delivery (HD): diagnosis, patient monitoring (PM), CT, diagnostic imaging tests, vaccine distribution (VD), and emergency medical services (EMS). The positive impact of 5G as a core technology for COVID-19 applications has enabled the exchange of huge data sets in Fangcang (cabin) hospitals and enhanced real-time CT with low latency. B5G-based cloud communication technologies not only serve as communication tools but also as CFI. B5G technology was developed to monitor vital signs (body temperature, heart rate, and peripheral blood oxygen saturation) affected by COVID-19. B5G telecommunications with mature IoMT is a core technology that can make key contributions to the provision of high-quality, sustainable healthcare to all patients. As a result, smart hospital care and remote diagnosis (SHCRD), DTCS, and ERCMR have been achieved. Wang et al. [19] integrate a B5G-enabled federated learning AI scheme to develop a robust auxiliary diagnostic model and serve as multiple institutions and a central cloud collaboration for the prevention, control, and treatment of COVID-19. B5G mobile networks can achieve high-speed medical data transmission, quick RT capabilities, and full-space connections of big doctor–patient medical data. The examination and monitoring of physiological signals can be achieved. The AI-based auxiliary diagnostic architecture includes three parts: a data collection layer, a diagnosis feedback layer, and a model cognition layer for doctor–patient with COVID-19 medical data. B5G is a significant tool that can quickly monitor the spread of this virus, recognize high-risk patients, and be useful for RT monitoring of this infection. The technical effectiveness of the B5G-based system includes CPCS, DTCS, HD, PM, SHCRD, CFI, and ERCMR. This study may also be instrumental in understanding and suggesting the development of a COVID-19 vaccine [20]. IoMT is a well-developed scheme of interconnected medical computing techniques and physical and mechanical medical devices that can transmit medical data over a specified network without any human intervention at any place and time. The advantages of 5G/B5G technologies with IoMT in the fight against COVID-19 include superior treatment, reduced mistakes, reduced expenses, effective control, and enhanced diagnosis. Blood pressure, body temperature, oxygen saturation, and pulse were monitored using B5G technology with IoMT. Whenever a vital sign crosses a threshold value (upper or lower limit), the IoMT system triggers an instant alert and sends a short message service (SMS) to the physician, informing them that the threshold value has been breached. The advantages of AI in the fight against COVID-19 include diagnosis, prediction, risk detection, fake news detection, drug development, understanding of the virus, early detection, and drug modeling. The technical effectiveness of the B5G-based IoMT system includes CPCS, DTCS, HD, PM, VD, SHCRD, CFI, and ERCMR.

Elmousalami et al. [21] demonstrated the impact of 5G infrastructure on human healthcare, the COVID-19 pandemic, and business. 5G can revolutionize healthcare against COVID-19 by providing better assistance to medical staff, RT monitoring of patients, analysis of monitored RT vital signs, and corrective decisions and diagnoses. 5G networks with AI and IoMT can improve medical efficiency during the COVID-19 epidemic. 5G-based thermal imaging (TI) enables real-time measurement of the temperature of moving bodies. Information on moving body temperature is accumulated in central monitoring devices based on 5G communication technology with high-speed data transmission and ultra-low latency. The technical effectiveness of the B5G-based IoMT system includes CPCS, HD, PM, SHCRD, CFI, and ERCMR.

BC-assisted UAV VD with RT monitoring and control using 6G-enhanced ultra-reliable low-latency communication was proposed [22]. CPCS, CT, CFI, and ERCMR can be achieved. The benefits of 5G technologies, implemented in healthcare and SWMDs, including patient health monitoring, continuous monitoring of chronic diseases, management of infectious diseases, and robotic surgery using 5G, are demonstrated [23]. 5G technology has a direct effect on clinical decision-making and can improve patient rehabilitation outside hospitals and continuously monitor human physical activity. Patient rehabilitation outside of hospitals (PROH), HD, PM, SHCRD, CFI, and ERCMR can be achieved. 5G-based mobile cardiovascular disease monitoring using SWMDs and deep learning schemes may effectively reduce COVID-19 mortality rates [24]. An RT streaming data processing framework (RTSDPF) was integrated to realize RT electrocardiography (ECG) signal data monitoring, analysis, and diagnosis. DTCS, HD, PM, and ERCMR can be achieved.

During and after the pandemic, effective measures must be taken to diagnose COVID-19 patients and mitigate the effects of the virus [25]. Emerging developments in IoMT, 6G wireless communication, and AI technology can be harnessed to combat COVID-19. The implementation of IoMT in hospitals enables highly integrated digital wireless transmission environments and RT clinical data collection, which can be used to identify clinical patterns, model risk interactions, and forecast effects using AI and deep learning systems. The cough sounds and chest X-ray images of patients with COVID-19 were analyzed using federated learning. DTCS, HD, PM, SHCRD, CFI, and ERCMR can be achieved. Intelligent reflector-viral detectors (IR-VD) have been developed for application in new public health concepts using a 6G mobile network [26]. 6G mobile communication systems can play an important role in detecting viruses shed by infected individuals. Intelligent reflective surface technology has been applied to reflect, refract, and diffract electromagnetic waves in the millimeter-wave and terahertz spectra. IR-VD with an array of patch antennas and adhesive viral detector strips was proposed for controlling beamforming and information encoding. Wireless indoor viral detection (VDE) was achieved. 

The IoMT provides the scalability required for this purpose, supporting continuous and reliable health monitoring of global public health during the COVID-19 outbreak [27]. A 5G-based IoMT healthcare platform provides the remote monitoring of patients in critical situations by integrating SWMDs to monitor patients with coronavirus disease in the ICU. IoMT can be adopted to collect and process patient medical data to promote rapid clinical interventions, while preventing contagion between clinical staff and infected patients. DTCS, HD, PM, SHCRD, and CFI can be achieved. There is an important need to develop sensitive online methods for the on-site diagnosis and monitoring of suspected COVID-19 patients [28]. To this end, a 5G-enabled fluorescence sensor (FS) for the quantitative detection of spike protein and nucleocapsid protein of COVID-19 using a mesoporous silica-encapsulated up-conversion nanoparticle (UCNPs@mSiO2) labeled lateral flow immunoassay is proposed. The proposed fluorescence sensor is IoMT-enabled and is accessible to edge hardware devices (personal computers, 5G smartphones, internet protocol television, etc.) through Bluetooth. Medical data can be transmitted to a 5G cloud server with ultra-low latency and high reliability for AI computation and analysis. HD, PM, and VDE can be achieved. Advanced 5G mobile communication services, such as telehealth and telemedicine, play a significant role in reducing the risk and fighting the spread of the COVID-19 pandemic and beyond [29]. CPCS, DTCS, HD, and PM can be achieved.

5G-based IoMT system with a cloud server is demonstrated. 5G-based mobile communication systems focus on 5G-empowered e-health and AI-based arrangements [30]. This chapter explores the use of AI and 5G technologies with self-diagnostic mechanisms to alleviate the spread of COVID-19. A cloud-based data analytics tool for mobile handsets is provided. 5G thermal imaging, 5G robots, and 5G IoMT have been integrated into cloud-based mobile platforms. CPCS, DTCS, HD, and PM can be achieved.

5G is not only proposed as a tool for communication between people but is also proposed as an instrument for connectivity between medical machines, in line with the implementation of IoMT [31]. Mobile medical networks are designed to generate a high level of connectivity, for which medical data must be contextualized, structured, and processed using AI in the cloud, requiring a storage infrastructure with large capacity, transmission equipment, and the development of algorithms and software for the treatment of large data. Mobile health (mHealth) involves the intensive use of sensors, AI, and IoMT to make remote diagnoses, monitor patients, and analyze special epidemiological situations by evaluating large volumes of medical data. CPCS, DTCS, HD, and PM can be achieved. Table 1 lists the technical features and technical effectiveness of 5G, B5G, and 6G during the fight against the COVID-19 pandemic.

## 3. Applications (Apps) Related to COVID-19

Anyanwu et al. [32] developed a mobile app (MAPP) (mobilMD) to promptly facilitate remote patient care and innovate the COVID-19-related hospital infrastructure (CRHI). MobilMD provided clinicians access to rapidly evolving institutional policies and protocols (IPP), facilitated remote patient care (FRPC), and gained widespread durable use at large academic medical centers. MobilMD focuses on RT, high-impact policy updates (HIPU) on patient care, such as personal protective equipment and ICU guidelines. MobilMD benefits from the innovation team’s understanding of the institutional culture and structure, resulting in shorter feedback loops for content and characteristic updates. 

Preventing infections, hospitalizations, intensive care treatments, and deaths (PIHICTD) can be achieved through smart hospital care (SHC) and CFI.

Kobayashi et al. [33] demonstrated how mobile messenger apps affect COVID-19 vaccine information (CVI). The Corowa-kun app provides instant and automated answers to 70 frequently asked COVID-19 vaccine questions using LINE in Japan. The apps identify vaccine hesitancy, assess risk factors, and investigate vaccine intentions (IVHRFIVI). 

IPP and HIPU can be achieved. CT apps have been developed to help contain or reduce the spread of COVID-19 [34]. The role of national culture in the acceptance of these apps has also been explored. The moderating role of national culture on the acceptability of CT apps in relation to power distance, individualism, long-term orientation, and indulgence in the pre-deployment phase was confirmed (PILIC). Quick and efficient CT via mobile apps is considered an important strategy for de-escalating lockdown measures (DELM). Public health institutions can increase the acceptance of COVID-19 CT apps. COVID-19 CT or proximity detection (CCTPD) is integrated into the apps. IPP and HIPU can be achieved. CT is a challenge for governments during the COVID-19 pandemic; Germany introduced a mobile-phone-based digital CT app [35]. The effectiveness of the app is analyzed; the insight concept associated with PIHICTD is demonstrated; and the infection cases prevented by the app are calculated using mathematical and statistical modeling (MSM). CCTPD is integrated into the apps. Awareness about COVID-19 (AC) and CFI can be achieved.

Kaiser et al. [36] developed a mobile-app-based intelligent portable healthcare system for employee-reported COVID-19 self-test data to detect possible suspects of COVID-19 infection. Data on employee health status (EHS) were established using the company’s database, and proximity and CT data were calculated by integrating the K-nearest neighbor and K-means techniques. The app tracks users’ proximity and traces contacts with other employees. This method can be applied to industrial factories and infectious disease detection. The app aids in prompting public health risk responses and management of the COVID-19 outbreak in the workplace, thus leading to a healthy work environment. Tele-monitoring (TM), TS, and CCTPD are integrated into the app. 

Detecting possible suspects for COVID-19 infection (DPSCI) and industrial application (IA) can be achieved.

The COVID-19 outbreak has evoked fear and anxiety among the public, and CT is a time-consuming and resource-intensive process [37]. A COVID-19 web app (WAPP) with symptom monitoring (SM) and CT records was also developed. CT details include demonstrated confirmed/suspected cases, individuals who have just returned from high-risk areas, and people who have been in contact for the past two weeks. A proactive approach to enhance current management strategies for COVID-19 has been elaborated. The SM functions of the WAPP include temperature, dry cough, shortness of breath, fatigue, muscle ache, and loss of sense of taste and smell. Travel history records travel destinations, dates, and returns. The electronic medical records of the patients visiting the healthcare institution are also shown. Personal health record (PHR), CCTPD, TS, and TM are integrated into the WAPP. The COVID-19 pandemic has limited face-to-face treatment in existing healthcare services. The effects of video-based telehealth services using a mobile PHR app for patients with metabolic risk factors have been investigated [38]. Healthcare professionals observed patients’ medical information using monitoring technology and mobile sensors (MS) and performed appropriate interventions. The effects of the services on changes in the patient’s metabolic risk factors were evaluated, and changes in the patient’s lifestyle and service satisfaction were analyzed. Changes in systolic and diastolic blood pressure, body weight, body mass index, waist circumference, fasting blood glucose, triglyceride, and high-density cholesterol levels were measured. The effectiveness of video-based TS supporting patients’ health status and lifestyle interventions using healthcare technologies, such as the mobile PHR app, TM, and RT video teleconsultation (VT), is integrated. Facilitating remote patient care (FRPC), AC, and SM can be achieved.

Patient experience with the Mawid app was evaluated during the COVID-19 pandemic in Al Hassa, Saudi Arabia [39]. The app was identified as easy to use, and users were highly satisfied with its services. The app allows patients to book, cancel, and/or reschedule their appointments at primary healthcare centers (BCRTAPHC) and manage their referral appointments. Users could assess the risk of COVID-19 transmission. Users were advised to enter their symptoms and travel details into an app for risk assessment. The app also helped users increase their awareness regarding COVID-19. The app is a CRHI, and CVI, PHR, TS, and TM are integrated. AC, SM, DPSCI, and CFI can be achieved. Smartphone apps with in situ recordings and MSs have been adopted for ecological momentary assessments [40]. Implementing such an app requires short development cycles (SDC) to react appropriately to abrupt changes during the pandemic. The Corona Health app was developed for questionnaire-based studies in combination with recordings from MSs. The Corona Health app is a viable tool for conducting research related to the COVID-19 pandemic and can be used as a blueprint for future emergency medical services (EMS)-based studies. The information collected by us will substantially improve our knowledge of mental and physical health states, traits, and trajectories, as well as their risk and protective factors against the COVID-19 pandemic and its diverse prevention measures. The technical features of the Corona Health app are situ recordings (SR) and appropriately react to abrupt changes in the pandemic (ARACP). FRPC, AC, DPSCI, and SM can be achieved. AI apps have been developed to achieve patient-centered wound care activities (PCWCA) and management from a clinician-and-patient perspective during the COVID-19 pandemic [41]. High-resolution cameras (HRCs) were used to capture wound images, and machine learning (ML) software was used to evaluate wound location, color, and exudate. These digital wound care documents were integrated into a cloud-based PHR system, and the wound application facilitated remote patient monitoring and maintained optimal wound care. When TM is integrated into the app, FRPC, DOSCI, and SM are achieved.

Mobile symptom-tracking apps are significant for monitoring the global pandemic crisis by providing near real-time, SR for medical and governmental responses [42]. A self-supervised ML scheme measuring information entropy was integrated into the COVIDCare app. The relevant stratifications of disease symptoms and the predictive potential of AI for extracting feature knowledge from medical records were explored. The app can aid the government in focusing on and complementing public health decisions. PHR, TS, and TM are adopted, and IPP, HIPU, and SM are achieved.

Wu et al. [43] systematically empirically investigated the factors affecting the adoption and evaluation of COVID-19-related apps. The factors both in the country and app that influence the adoption and evaluation of COVID-19 apps were investigated. A total of 267 COVID-19 apps in the App Store and Google Play were collected. The functions of these apps included providing health information, CT, home monitoring, and consultation (CONSU). The technical features of the apps are CCTPD, PHR, TS, and TM. The technical effectiveness of the apps includes FRPC, CT, and SM. Getz et al. [44] developed a WAPP to explicitly identify susceptible, contact, latent, asymptomatic, symptomatic, and recovered classes of individuals, as well as a parallel set of response classes, subject to lower pathogen-contact rates. The comma-separated values files of the incidence and mortality rate data were entered into the WAPP. The maximum-likelihood estimation (MLE) method is integrated into a WAPP, and the WAPP utilizes TS, TM, and PHR. AC, DPSCI, and SC are achieved. An Android-based health promotion (healthcare) app for clinical cardiovascular diseases has been proposed [45]. The mobile app (MAPP) can provide health promotion recommendations to physicians through mobile cloud-based health promotion. These real-time recommendations are based on users’ systolic and diastolic blood pressure, pulse, sleep time, ECG, climate, diet, and movement records, utilizing remote video conferences. The cloud-based blood pressure and electrocardiogram healthcare modules, remote video technique, video recording module, message module, and cloud-based climate, diet, and movement module were integrated into the mobile app. This mobile health promotion (MHP) app can be used to manage various chronic and cardiovascular diseases, anywhere and at any time. The app was designed to improve cost, time, efficiency, health management and monitoring, and quality of care. TS, TM, PHR, VT, and CONSU are used in the MAPP, and RT and SM are achieved. Table 2 presents the technical features and technical effectiveness of the apps for the fight against the COVID-19 pandemic.

## 4. Discussions

Table 3 lists the technical features of 5G systems. In 5G, millimeter-wave (mmWave) communications can be achieved, and the technical features of 5G include an explosion in the number of connected devices, a large diversity of use cases and requirements, massive multi-input multi-output (MIMO), and massive increases in data volumes and rates. 5G communication techniques must connect billions of smart devices, such as surveillance cameras, smart home/grid devices, and connected medical sensors. 5G-based wireless connections for at least 100 billion devices and 10 Gb/s delivered to individual patients can be achieved [46]. Mass low-latency and ultra-reliable 5G connectivity has been established among patients, medical machines, and devices, which will ultimately lead to the era of the IoMT for patients. SpO2, body temperature, blood pressure, pulse, digital X-ray images, respiratory rate, heart rate, ECG, EEG, and audio and video physiological parameters can be monitored using these 5G-based wireless medical machines, devices, and sensors. The transmission bit error rates of these physiological parameters must be $10^{-7}$ or below.

Table 4 presents the technical features of the B5G system. B5G technology enables 5G to achieve higher data rates, lower latency, greater capacity, and more efficient spectrum utilization. More efficient networks, new services, ecosystems, and revenues can be provided. Enhanced mobile broadband (eMBB), ultra-reliable low-latency communications (URLLC), massive machine-type communications (mMTC), and enhanced vehicle to everything (eV2X) can be achieved. These advanced B5G technical features can be integrated into B5G-based IoMT systems.

Table 5 lists the technical features of the 6G network. The 5G technology is connected to things, and the 6G scheme is connected to intelligence and ubiquitous wireless intelligence. In the 6G era, terahertz (THz) communication at T bits per second (Tbs) was delivered to individual patients. The technical features of 6G include super-massive MIMO, holographic beamforming (HBF), orbital angular momentum (OAM) multiplexing, laser communication, visible-light communication (VLC), BC-based spectrum sharing, quantum computing, reconfigurable intelligent surfaces, BC, high-capacity backhaul connectivity, cloud-fog architecture, machine-type communications, edge intelligence, and pervasive AI. 6G-based intelligent communication networks adopt cell-less architectures to enable ubiquitous three-dimensional (3D) coverage (low-earth-orbit (LEO) satellites, land-based mobile cellular, and underwater) communication networks.

6G technology can achieve mobile healthcare, telesurgery, 6G-based wireless brain–computer interaction (BCI) connections to medical machines, devices, and sensors, as well as large intelligent service (LIS). Mixed reality (MR) medical applications involving real-time patient interaction in immersive environments can be realized. The transmission data rate was 1 Tbps, and the characteristics of the mobile connections were minimum latency and ultra-high reliability (UHR). Medical holographic telepresence (MHT) applications that can synchronize various viewing angles have been proposed. The transmission data rate was 4.32 Tbps, and the characteristics of mobile connections were submillisecond latency and UHR. Before surgery, MR and MHT schemes can be integrated into informed patient consent procedures, allowing patients to understand the surgical procedures and risks in detail. The MR and MHT schemes can also be integrated into medical and health education programs to prevent diseases related to viral infections. 

Table 6 lists the overview of advanced 5G/B5G/6G technical features and effectiveness during the fight against the COVID-19 pandemic era. The technical features and effectiveness of 5G/B5G/6G are IoMT, AI, CPCS, CT, CFI, ERCMR, HD, PM, RT, and SHCRD. 5G technology is based on connected things, and the 6G scheme is based on connected intelligence and ubiquitous wireless intelligence. 5G-based wireless connections are low-latency and ultra-reliable for at least 100 billion devices, and 10 Gb/s can be delivered to individual patients. 6G-based wireless connections have submillisecond latency and UHR and can deliver Tbs to individual patients. The 6G-based IoMT system has higher transmission data rates, lower transmission latency, and UHR. MR, MHT, and LIS schemes can be integrated into 6G-based IoMT systems. The technical features and effectiveness of 5G and B5G include DTCS and cloud applications. The technical features and effectiveness of 5G include the UAV, robots, SWMD, BC, VR, video, DPPICUCP, TI, TS, RTSDPF, FS, ICP, PROH, VDE, telemedicine, and line. The technical features and effectiveness of B5G are the VD, EMS, and SMS, respectively. The technical features and effectiveness of 6G include UAV, VD, IR-VD, M/T Hz, and wireless indoor VDE.

Table 7 presents an overview of advanced apps’ technical features and effectiveness during the fight against the COVID-19 pandemic era. The technical features and effectiveness of MAPP and CRHI include FRPC, IPP, HIPU, RT, PIHICTD, SHC, CFI, CVI, AC, TM, TS, DPSCI, PHR, and SM. The technical features and effectiveness of MAPP include the CT, line, IVHRFIVI, CCTPD, MSM, DELM, EHS, IA, VT, MS, SR, SDC, PCWCA, CONSU, PILIC, K-nearest neighbor and K-means, ARACP, ML, cloud, and wound images. The technical features and effectiveness of CRHI include only WAPP and MLE.

## 5. Conclusions

The COVID-19 pandemic and its aftermath have raised challenging research questions across multiple areas to mitigate its impact on human life. In this paper, studies on 5G, B5G, 6G, advanced communication technologies, and advanced mobile apps to combat the COVID-19 outbreak are presented. Fifteen papers associated with the concepts of translational informatics, prevention and treatment of viral infections, 5G/B5G/6G mobile communication techniques, and IoMT were elaborated. Sixteen papers associated with the concepts of advanced 5G, B5G, and 6G mobile communication technologies with applications regarding COVID-19 were illustrated. Fourteen papers associated with the concept of apps related to COVID-19 were discussed. 

The technical features of 5G, B5G, and 6G during the fight against the COVID-19 pandemic included IoMT, UAV, SWMD, IR-VD, FS, BC, RTSDPF, AI, VR, SMS, TI, and M/T. The technical effectiveness of 5G, B5G, and 6G during the fight against the COVID-19 pandemic included CPCS, DTCS, DPPICUCP, HD, PM, CT, VD, EMD, SHCRD, CFI, RT, PROH, ERCMR, and VD. 

The technical features of the apps for the fight against the COVID-19 pandemic included MAPP, CVI, CCTPD, VT, TS, PHR, TM, BCRTAPHC, SR, MS, SDC, ARACP, MSM, AI, CONSU, and WAPP. The technical effectiveness of the apps for the fight against the COVID-19 pandemic followed the order: FRPC, IPP, HIPU, RT, IVHRFIVI, PILIC, DELM, PIHICTD, DPSCI, EHS, IA, SM, AC, and PCWCA. These technical features and effectiveness highlight the innovative design concept of advanced 5G/B5G/6G-based information communication techniques for the prevention of infections, diagnosis, and treatment of rapidly outbreaking virus-associated diseases.

## Figures and Tables

**Table 1:** Technical features and technical effectiveness of 5G, B5G, and 6G during the fight against the COVID-19 pandemic.

References	Technical Features	Technical Effectiveness
Siriwardhana et al. [15]	1. 5G; 2. IoMT; 3. SWMD.	1. RT; 2. TS; 3. CT; 4. ICP.
Chamola et al. [16]	1. 5G; 2. IoMT; 3. UAV; 4. robots; 5. SWMD; 6. BC; 7. AI.	1. CPCS; 2. DTCS; 3. CFI; 4. ERCMR.
He et al. [17]	1. 5G; 2. VR; 3. Video; 4. SWMD.	1. CPCS; 2. DTCS; 3. DPPICUCP; 4. CFI; 5. ERCMR.
Moglia et al. [18]	1. B5G; 2. IoMT; 3. cloud.	1. DTCS; 2. HD; 3. PM; 4. CT; 5. VD; 6. EMS; 7. SHCRD; 8. CFI; 9. ERCMR.
Wang et al. [19]	1. B5G; 2. AI; 3. cloud.	1. CPCS; 2. DTCS; 3. HD; 4. PM; 5. SHCRD; 6. CFI; 7. ERCMR.
Muhammad et al. [20]	1. B5G; 2. IoMT; 3. AI; 4. SMS.	1. CPCS; 2. DTCS; 3. HD; 4. PM; 5. VD; 6. SHCRD; 7. CFI; 8. ERCMR.
Elmousalami et al. [21]	1. 5G; 2. IoMT; 3. AI; 4. TI.	1. DTCS; 2. HD; 3. PM; 4. SHCRD; 5. CFI; 6. RT; 7. ERCMR.
Verma et al. [22]	1. 6G; 2. UAV.	1. CPCS; 2. CT; 3. VD; 4. CFI; 5. RT; 6. ERCMR.
Devi et al. [23]	1. 5G; 2. robots; 3. SWMD.	1. HD; 2. PM; 3. SHCRD; 4. CFI; 5. PROH; 6. ERCMR.
Tan et al. [24]	1. 5G; 2. SWMD; 3. RTSDPF; 4. AI.	1. DTCS; 2. HD; 3. PM; 4. RT; 5. ERCMR.
Muhammad et al. [25]	1. 6G; 2. IoMT; 3. AI.	1. DTCS; 2. HD; 3. PM; 4. SHCRD; 5. CFI; 6. RT; 7. ERCMR.
Šiljak et al. [26]	1. 6G; 2. IR-VD; M/T Hz.	wireless indoor VDE.
Barroca Filho et al. [27]	1. 5G; 2. IoMT; 3. SWMD.	1. DTCS; 2. HD; 3. PM; 4. SHCRD; 5. CFI.
Guo et al. [28]	1. 5G; 2. IoMT; 3. FS; 4. AI; 5. cloud.	1. HD; 2. PM; 3. VDE.
Hussein et al. [29]	1. 5G; 2. TS; 3. telemedicine.	1. CPCS; 2. DTCS; 3. HD; 4. PM.
Ahmed et al. [30]	1. 5G; 2. IoMT; 3. robots; 4. AI; 5. cloud; 7. TI.	1. CPCS; 2. DTCS; 3. HD; 4. PM.
Solleiro et al. [31]	1. 5G; 2. IoMT; 3. AI; 4. line.	1. CPCS; 2. DTCS; 3. HD; 4. PM.

**Table 2:** Technical features and technical effectiveness of the apps for the fight against the COVID-19 pandemic.

References	Technical Features	Technical Effectiveness
Anyanwu et al. [32]	1. MAPP; 2. CRHI.	1. FRPC; 2. IPP; 3. HIPU; 4. RT; 5. PIHICTD; 6. SHC; 7. CFI.
Kobayashi et al. [33]	1. MAPP; 2. CVI; 3. line.	1. IPP; 2. HIPU; 3. IVHRFIVI.
Dzandu et al. [34]	1. MAPP; 2. CCTPD.	1. IPP; 2. HIPU; 3. PILIC; 4. DELM; 5. CT.
Ellmann et al. [35]	1. MAPP; 2. CCTPD; 3. MSM.	1. AC; 2. PIHICTD; 3. CT; 4. CFI.
Kaiser et. al. [36]	1. CCTPD; 2. TS; 3. TM; 4. K-nearest neighbor and K-means.	1. AC; 2. CT; 3. DELM; 4. DPSCI; 5. EHS; 6. IA.
Yap et. al. [37]	1. CCTPD; 2. PHR; 3. TS; 4. TM; 5. WAPP.	1. CT; 2. SM.
Park et al. [38]	1. MAPP; 2. VT; 3. PHR; 4. TS; 5. TM; 6. MS.	1. FRPC; 2. AC; 3. RT; 4. SM.
AlAli et al. [39]	1. MAPP; 2. CRHI; 3. CVI; 4. PHR; 5. TS; 6. TM; 7. BCRTAPHC.	1. AC; 2. SM; 3. DPSCI; 4. CFI.
Beierle et al. [40]	1. MAPP; 2. SR; 3. MS; 4. SDC; 5. ARACP.	1. FRPC; 2. AC; 3. RT; 4. EMS; 5. DPSCI; 6. SM.
Barakat-Johnson et al. [41]	1. MAPP; 2. HRC; 3. PHR; 4. TM; 5. ML; 6. cloud.	1. FRPC; 2. PCWCA; 3. wound images; 4. DOSCI; 5. SM.
Sousa et al. [42]	1. MAPP; 2. PHR; 3. TS; 4. TM; 5. SR; 6. ML.	1. IPP; 2. HIPU; 3. SM.
Wu et al. [43]	1. MAPP; 2. CCTPD; 3. PHR; 4. TS; 5. TM; 6. CONSU.	1. FRPC; 2. CT; 3. SM.
Getz et al. [44]	1. WAPP; 2. TS; 3. TM; PHR; 4. MLE.	1. AC; 2. SM; 3. DPSCI.
Lin et al. [45]	1. MAPP; 2. VT; 3. PHR; 4. TS; 5. TM; 6. CONSU; 7. cloud.	1. MHP; 2. RT; 3. SM.

**Table 3:** Technical features of 5G systems.

Technical Features	
Millimeter-wave (mmWave) communications.	An explosion in the number of connected devices.
Large diversity of use cases and requirements.	Massive increase in data volumes and rates.
Connect billions of smart devices, such as surveillance cameras, smart-home/grid devices, and connected sensors.	5G-based wireless connections for at least 100 billion devices, and 10 Gb/s delivered to individual patients.
Mass low-latency and ultra-reliable 5G connectivity has been established among patients, medical machines, devices, and sensors, which will ultimately lead to patients in the era of the IoMT.	Massive MIMO.

**Table 4:** Technical features of B5G.

Technical Features	
Make 5G capable of achieving higher data rates, lower latency, greater capacity, and more efficient spectrum utilization.	Significantly much more efficient networks, new services, new ecosystems, and new revenues can be provided.
eMBB.	URLLC.
mMTC.	eV2X.

**Table 5:** Technical features of 6G.

Technical Features					
Connected intelligence.			Ubiquitous wireless intelligence.		
THz communications.			Super-massive MIMO.		
HBF.			OAM multiplexing.		
Laser communication.			VLC.		
BC-based spectrum sharing.			Quantum computing.		
Cell-less architectures to enable ubiquitous 3D coverage (LEO satellite, land-based mobile cellular, and underwater) intelligent communication networks.					
Reconfigurable intelligent surface.			BC.		
Tbs delivered to individual patients.			High-capacity backhaul connectivity.		
Cloud-fog architecture.			Machine-type communications.		
Edge intelligence.			Pervasive AI.		
MR medical applications with real-time patients interaction in an immersive environment.			MHT application synchronizing many viewing angles.		
1 Tbps	Minimum latency.	UHR	4.32 Tbps	Sub-ms latency	UHR
Telesurgery.			Mobile healthcare.		
6G-based wireless BCI connections to medical machines, devices, and sensors.			LIS.		

**Table 6:** Overview of advanced 5G/B5G/6G technical features and effectiveness during the fight against the COVID-19 pandemic era.

	Technical Features
5G [15,16,17,21,23,24,27,28,29,30,31]	IoMT, UAV, robots, SWMD, BC, AI, CPCS, DTCS, CT, CFI, ERCMR, VR, video, DPPICUCP, TI, TS, HD, PM, SHCRD, RT, RTSDPF, cloud, FS, ICP, PROH., VDE, telemedicine, line.
B5G [18,19,20]	IoMT, cloud, DTCS, HD, PM, CT, VD, EMS, SHCRD, CFI, ERCMR, AI, CPCS, SMS, RT.
6G [22,25,26]	IoMT, UAV, CPCS, CT, VD, CFI, RT, ERCMR, AI, HD, PM, SHCRD, IR-VD, M/T Hz, wireless indoor VDE.

**Table 7:** Overview of advanced apps’ technical features and effectiveness during the fight against the COVID-19 pandemic era.

	Technical Features
MAPP [32,33,34,35,36,38,39,40,41,42,43,45]	FRPC, IPP, HIPU, RT, CVI, CT, line, IVHRFIVI, PIHICTD, CCTPD, MSM, AC, TS, TM, DELM, DPSCI, EHS, IA, VT, PHR, MS, SM, BCRTAPHC, SR, SDC, PCWCA, CONSU, PILIC, SHC, CFI, K-nearest neighbor and K-means, ARACP, ML, cloud, wound images.
CRHI [32,39,44]	FRPC, IPP, HIPU, RT, PIHICTD, SHC, CFI, CVI, AC, PHR, TM, TS, BCRTAPHC, DPSCI, SM, WAPP, MLE.

## Data Availability

Not applicable.

## Abbreviations

AC	awareness about COVID-19
apps	applications
AI	artificial intelligence
ARACP	appropriately react to abrupt changes in the pandemic.
BC	blockchain
BCI	brain–computer interaction
BCRTAPHC	book, cancel, and/or reschedule their appointments at primary healthcare centers
B5G	beyond 5G
CCTPD	COVID-19 contact-tracing or proximity detection
DELM	de-escalate lockdown measures
DPSCI	detecting possible suspects for COVID-19 infection
CFI	core fundamental infrastructure
COVID-19	coronavirus disease 2019
CONSU	consultation
CPCS	combat and prevention COVID-19 strategies
CT	contact tracing
CVI	COVID-19 vaccine information
CR	cardiac rehabilitation
CRHI	COVID-19-related hospital infrastructure
DTCS	diagnosis and treatment COVID-19 strategies
DPPICUCP	decreasing the psychological problems of ICU COVID-19 patients
ECG	electrocardiography
EEG	electroencephalogram
EHS	employees’ health status
eMBB	enhanced mobile broadband
EMS	emergency medical services
ERCMR	effectively reducing COVID-19 mortality rates
eV2X	enhanced vehicle to everything
4G	fourth generation
5G	fifth generation
FRPC	facilitate remote patient care
FS	fluorescence sensor
HD	healthcare delivery
HBF	holographic beamforming
HHT	Hilbert–Huang transformation
HIPU	high-impact policies update
IA	industrial application
ICU	intensive care unit
ICP	immediate control policies
LEO	low-earth-orbit
LIS	large intelligent service
IoT	internet of things
IoMT	internet of medical things
IPP	institutional policies and protocols
IR-VD	intelligent reflector-viral detectors
IVHRFIVI	identify vaccine hesitancy, assess risk factors, and investigate vaccine intention
LTE	long-term evolution
MAPP	mobile app
MHP	mobile health promotion
MHT	medical holographic telepresence
MIMO	multi-input multi-output
ML	machine learning
MLE	maximum-likelihood estimation
mMTC	massive machine-type communications
mmWave	millimeter-wave
M/THz	mmwave/terahertz
MR	mixed reality
MS	mobile sensors
MSM	mathematical and statistical modeling
NR	new radio
OAM	orbital angular momentum
PCWCA	patient-centered wound care activities.
PHR	personal health record
PIHICTD	preventing infections, hospitalizations, intensive care treatments, and deaths
PILIC	power distance, individualism, long-term orientation, and indulgence in the pre-deployment phase are confirmed
PM	patient monitoring
PROH	patient rehabilitation outside of hospitals
RT	real-time
RTSDPF	RT streaming data processing framework
6G	sixth generation
SDC	short development cycles
SHC	smart hospital care
SHCRD	smart hospital care, and remote diagnosis
SM	symptom monitoring
SMS	short message service
SR	situ recordings
SWMD	smart wearable medical devices
Tbs	T bits per second
3D	three dimension
THz	terahertz
TI	thermal imaging
TM	tele-monitoring
TS	telehealth services
UAVs	unmanned aerial vehicles
UHR	ultra-high reliability
URLLC	ultra-reliable low-latency communications
VD	vaccine distribution
VDE	viral detection
VLC	visible-light communication
VR	virtual reality
VT	video teleconsultation
WAPP	web app
WHO	World Health Organization
